# 4.3 ENHANCING SOCIAL FUNCTIONING AND LONG-TERM RECOVERY IN YOUNG PEOPLE WITH FIRST EPISODE PSYCHOSIS (FEP) AND YOUNG PEOPLE AT ULTRA HIGH RISK (UHR) FOR PSYCHOSIS: A NOVEL ONLINE SOCIAL THERAPY APPROACH

**DOI:** 10.1093/schbul/sby014.010

**Published:** 2018-04-01

**Authors:** Mario Alvarez-Jimenez, John F Gleeson, Simon Rice, Sarah Bendall, Simon D’Alfonso, Dina Eleftheriadis, Daniela Cagliarini, Penni Russon, Patrick D McGorry, Barnaby Nelson

**Affiliations:** 1 Orygen, The National Centre of Excellence in Youth Mental Health, The University of Melbourne; 2 Australian Catholic University, School of Psychology; 3 The University of Melbourne

## Abstract

**Background:**

Specialized early intervention services have demonstrated improved outcomes in first episode psychosis (FEP); however, functional recovery lags behind symptomatic remission, and many FEP patients remain socially isolated with poor functional outcomes. Similarly, psychological and pharmacological treatments have been demonstrated to reduce rates of transition to psychosis in Ultra High Risk (UHR) patients. However, recent research shows that UHR patients have a poor functional outcome regardless of transition to psychosis. These findings have resulted in widespread calls for new treatments aimed at improving functioning in both FEP and UHR patients.

The aim of these studies was to determine the safety, acceptability, feasibility and treatment effects of an advanced online social media based intervention specifically designed to enhance social functioning in FEP and UHR patients.

**Methods:**

Our multi-disciplinary team of 35 researchers, software engineers, professional writers, clinical psychologists, comic developers, experts in human-computer interaction and young people has developed novel online social media platforms for young people with FEP (Horyzons), and UHR patients (Momentum). Our interventions integrate: i) peer-to-peer social networking, ii) tailored therapeutic interventions, iii) expert and peer-moderation, and iv) new models of psychological therapy (strengths-based models, self-compassion and mindfulness). The acceptability and safety of these platforms have been evaluated through 2 pilot studies in FEP (N=20; 1 month intervention), and UHR (N=15; 2 months intervention). In addition, the effectiveness of Horyzons is currently being evaluated in a large 5 year RCT in FEP (N=170; 18 months intervention).

**Results:**

UHR pilot: System usage was high, with a total 270 logins (18/user), 749 posts (58/user), 170 therapy modules completed (12/user), and 67% of users being actively engaged over the trial. All participants reported a positive experience using Momentum and would recommend it to others. 93% considered Momentum to be helpful. Analysis revealed a significant increase in social functioning (p<0.001; d=2.39) and satisfaction with life (p=0.03; d=0.48) at follow-up. There was a significant increase in therapy mechanisms directly targeted by Momentum including strengths usage (p=0.03; d=0.46), mindfulness skills (p=0.04; d=0.36) and components of social support. There were significant correlations between system usage and improvements in social functioning (r=0.63 p=0.02), social support (r=0.62 p=0.02) and strengths usage (r=0.51 p=0.06).

FEP pilot: system use was high. The majority of FEP participants reported feeling safe (100%) and more socially connected (60%) using Horyzons. There was a significant reduction (d=0.60; p=0.03) in depressive symptoms at follow-up.

FEP RCT: Horyzons’ safety outcomes have been consistently strong. System usage is being high, with an average 101 logins, 70 posts, and 11 therapy modules per user, and 60% of users being engaged with the online system for a period of 18 months.

**Discussion:**

Horyzons and Momentum are the first online interventions designed to improve functional outcomes in FEP and UHR patients. Momentum is engaging, safe, may improve social functioning and satisfaction with life in UHR patients and appeared to specifically improve therapeutic mechanisms directly targeted by the online intervention. Horyzons is safe and engaging (over prolonged periods of time) and may improve depression and social connectedness in FEP patients.

